# Offering mailed nicotine replacement therapy and Quitline support before elective surgery: a randomised controlled trial

**DOI:** 10.5694/mja2.51453

**Published:** 2022-03-10

**Authors:** Ashley R Webb, Lisa Coward, Darshana Meanger, Samuel Leong, Sarah L White, Ron Borland

**Affiliations:** ^1^ Peninsula Health Melbourne VIC; ^2^ Monash University Melbourne VIC; ^3^ Quit Victoria Melbourne VIC; ^4^ The University of Melbourne Melbourne VIC

**Keywords:** Smoking, Prehospital care, Treatment outcome, Hospital medicine, Delivery of healthcare, Education, public health, Health services research, Health promotion, Preventive health services, Perioperative care

## Abstract

**Objective:**

To assess whether offering free mailed nicotine replacement therapy (NRT) and telephone counselling to smokers on elective surgery waiting lists increases quitting before surgery.

**Design, setting:**

Randomised, controlled trial at Frankston Hospital, a public tertiary referral hospital in Melbourne.

**Participants:**

Adult smokers added to elective surgery waiting lists for operations at least ten days in the future, 1 April 2019 ‒ 3 April 2020.

**Intervention:**

In addition to normal care, intervention participants received a brochure on the risks of low frequency smoking, an offer of Quitline call‐back registration, and an offer of mailed NRT according to reported daily smoking: 1‒9 cigarettes/day, 2 mg lozenges; 10‒15/day, 7‒14 mg patches [three weeks] and 2 mg lozenges; > 15/day, 7‒21 mg patches [five weeks] and 2 mg lozenges.

**Main outcome measures:**

Primary outcome: quitting at least 24 hours before surgery, verified by exhaled carbon monoxide testing. Secondary outcomes: quitting at least four weeks before surgery, adverse events, and (for those who had quit before surgery) abstinence three months after surgery.

**Results:**

Of 748 eligible participants (control, 363; intervention, 385), 516 (69%) had undergone elective surgery when the trial was terminated early (for COVID‐19‐related reasons) (intervention group, 274; control group, 242). 122 of the 385 intervention participants (32%) had accepted the offer of cessation support. The proportions of intervention participants who quit at least 24 hours before surgery (18% *v* 9%; odds ratio [OR], 1.97; 95% CI, 1.22‒3.15) or at least four weeks before surgery (9% *v* 4%; OR, 2.20; 95% CI, 1.08–4.50) were larger than for the control group. Three months after surgery, 27 of 58 intervention (47%) and 12 of 25 control participants (48%) who quit before surgery reported not smoking in the preceding seven days. No major adverse events were reported.

**Conclusion:**

Uptake of free mailed NRT and Quitline support by smokers on elective surgery waiting lists was good, and offering additional support was associated with higher proportions of smokers quitting before surgery.

**Trial registration:**

Australian New Zealand Clinical Trials Registry, ACTRN12619000032156 (prospective).

**The known**: Quitting smoking four weeks or more before surgery reduces peri‐operative risks. Nicotine replacement therapy and telephone counselling are effective, but in Australia are not routinely offered to smokers before elective surgery.

**The new**: Cessation help was accepted by almost one‐third of smokers to whom it was offered. Quitting attempts prior to surgery were more frequent among those who received the offer than those who did not, and the proportion who quit was about twice as large.

**The implications**: Systematically offering cessation support was a low cost means for encouraging elective surgery patients to quit. We recommend it to health services to overcome variability in cessation support provided by clinicians.

The risks of wound infections, cardiopulmonary complications, and higher health care costs are greater for smokers than non‐smokers undergoing elective surgery,^1^ but can be reduced if people quit at least four weeks before surgery.^2^ Although Australian and New Zealand colleges of surgery and anaesthesia recommend that clinicians help smokers to quit,^3^, ^4^ many undergo elective surgery without being offered help.^5^, ^6^ Evidence‐based therapies, including nicotine replacement therapy (NRT)^7^ and telephone counselling,^8^ could be routinely offered by health services when listing people for elective surgery; the subsequent waiting period (typically several weeks or months) provides an opportunity to help smokers stop smoking, reduce peri‐operative risks, and achieve longer term health benefits.

Our randomised, controlled trial examined whether offering free mailed NRT and Quitline referral to smokers on elective surgery waiting lists increased the proportion who quit smoking before surgery.

## Methods

We undertook a randomised, controlled trial at Frankston Hospital (Peninsula Health, Melbourne), a public tertiary hospital offering most surgical specialties apart from cardiac and neurosurgery, during 1 April 2019 – 3 April 2020. In 2015, 17.2% of adults in the Frankston local government area smoked every day.^9^ The trial was prospectively registered with the Australian New Zealand Clinical Trials Registry (ACTRN12619000032156; 11 January 2019).

Research assistants identified smokers by screening responses to tobacco use questions in the standard hospital waiting list health questionnaire. All adult smokers added to the elective surgery waiting list were enrolled in the trial, except people allergic to NRT, known to be pregnant or breastfeeding, weighing less than 45 kg, or unable to understand the study requirements. People already using pharmaceutical smoking cessation aids were not offered NRT, but were not excluded from group allocation. People undergoing endoscopy or surgery within ten days of listing (typically category 1 [urgent] surgery) were not enrolled. Our major analyses were restricted to people who underwent surgery at Frankston Hospital by 31 July 2020.

### Intervention and study procedures

Smokers were randomised (1:1) to the control or intervention groups using simple computer‐generated sequences (www.randomizer.org). Planned stratification by smoking frequency was not undertaken (Supporting Information, part 1). Participants were assigned sequential study numbers and entered into an Excel (Microsoft) database including a concealed column for group allocation. All were provided standard care (a brochure on smoking and surgery^10^). Intervention group smokers also received a printed offer of free mailed NRT and Quitline support (Supporting Information, part 2), and a study‐specific brochure on the risks of low frequency smoking (fewer than ten cigarettes per day) was mailed to light or intermittent smokers (Supporting Information, part 3). Participants in neither group were informed that the hospital was providing some smokers extra support or that they were participating in a research trial.

Intervention group participants were asked to contact us by telephone, text, or email to initiate cessation support, and we telephoned them once (at seven days) if they did not (messages left for unanswered calls). Those who accepted the offer of support were offered registration with the Quitline call‐back service and posted nicotine substitution products (Nicotinell, Perrigo):

▪ low frequency smokers (1–9 cigarettes/day): 72 × 2 mg lozenges;
▪ intermediate frequency smokers (10–15 cigarettes/day): two weeks’ supply of 14 mg patches, one week’s supply of 7 mg patches, and 72 × 2 mg lozenges;
▪ high frequency smokers (more than 15 cigarettes/day): three weeks’ supply of 21 mg patches, one week’s supply each of 14 mg and 7 mg patches, and 72 × 2 mg lozenges.

Research assistants phoned all participants about their NRT use and adverse events one and five weeks after enrolment; adverse events could also be reported to a dedicated mobile number. Research assistants blinded to allocation administered a questionnaire on smoking and quitting behaviour since enrolment to participants listed on daily hospital operation lists (Supporting Information, part 4); non‐responders were classified as current smokers. People who claimed to have quit at least 24 hours before surgery were tested with a piCO+ Smokerlyzer (Bedfont Scientific). An expired carbon monoxide value of 8 parts per million (ppm) or less was deemed to confirm cessation;^11^ people who refused testing were classified as current smokers.

In an exploratory sub‐study, a relapse prevention kit (20 × 2 mg nicotine lozenges, printed relapse prevention advice) was offered on the day of surgery to all people who had quit smoking, and we telephoned them three months after their operation about self‐reported abstinence during the preceding seven days.

### Outcomes

The primary outcome was the proportion of smokers who quit at least 24 hours before surgery, confirmed by carbon monoxide analysis. As Frankston Hospital banned carbon monoxide testing in March 2020 (Supporting Information, part 1), we added the unplanned analysis of reported quitting at least 24 hours before surgery, confirmed by carbon monoxide testing when possible. Secondary outcomes were reported quitting four or more weeks prior to surgery (confirmed by carbon monoxide testing on day of surgery), quitting activity during waiting period (successful attempts, and unsuccessful attempts sustained for more than 24 hours), cessation medication use and Quitline contacts during the waiting period, and abstinence three months after surgery (for smokers who quit before surgery). Smokers who reported amount smoked in the health questionnaire and did not quit at least 24 hours before surgery were asked on the day of surgery about amount smoked during the waiting period; it was assumed that smoking was not reduced for participants with expired carbon monoxide values exceeding 8 ppm or those who refused testing.

### Statistical analysis

Based on our pilot study finding of control group abstinence on admission of about 10%,^12^ we estimated that 199 people per group were required to detect a ten percentage point difference in the proportions of control and intervention participants who quit smoking before surgery (power, 80%; α = 0.05). We aimed to recruit at least 20% more participants to allow for the dropout of participants who did not undergo surgery during the study period. We undertook an intention‐to‐treat analysis by assigned group; associations between intervention and quitting are reported as odds ratios (ORs) with 95% confidence intervals (CIs). Statistical analyses were conducted in Stata 13.0.

### Ethics approval

The Peninsula Health Human Research Ethics Committee approved the study, and waived the requirement for consent by participating patients (HREC/49484/PH‐2019).

## Results

Of the 762 enrolled participants, 748 were eligible for the study (fourteen people who were not current smokers had ticked the health questionnaire “smoker” box in error) (Box 1), of whom 516 (69%) had undergone elective surgery at Frankston Hospital by 31 July 2020 (intervention group, 274; control group, 242) (Box 2). The median time from listing to surgery was 114 days (interquartile range [IQR], 53–203 days) for the control group and 99 days (IQR, 56–194 days) for the intervention group. The local impact of coronavirus disease 2019 (COVID‐19) led to the study being terminated early, on 7 August 2020, at which point 193 eligible participants (intervention group, 89; control group, 104) had not yet undergone surgery at Frankston Hospital (26%) (Supporting Information, part 1).

Box 1 **Baseline characteristics of 748 eligible participants at time of listing for elective surgery, based on responses to pre‐surgery health questionnaire**

**Table:** 

Characteristic	Control group	Intervention group
Participants	363	385
Sex (women)	210 (58%)	221 (57%)
Age (years), mean (SD)	50.1 (15.6)	49.6 (15.1)
Cigarettes per day, mean (SD)	12.3 (7.1)	12.7 (7.8)
Not reported	52 (14.3%)	42 (10.9%)
Surgery category		
1 (within 30 days)	30 (8%)	29 (8%)
2 (within 60 days)	237 (65%)	250 (65%)
3 (within 12 months)	96 (26%)	106 (28%)
Surgery type		
General surgery	92 (25%)	86 (22%)
Gynaecology	79 (22%)	89 (23%)
Orthopaedic	67 (18%)	66 (17%)
Urology	43 (12%)	41 (11%)
Plastic surgery	35 (10%)	22 (6%)
Vascular	27 (7%)	45 (12%)
Ear/nose/throat	20 (6%)	35 (9%)
Thoracic	0	1 (0.3%)

SD = standard deviation.

Box 2 **Selection, randomisation, screening, and participation of 762 people placed on waiting lists for elective surgery at Frankston Hospital, 1 April 2019 ‒ 3 April 2020**

**Table:** 

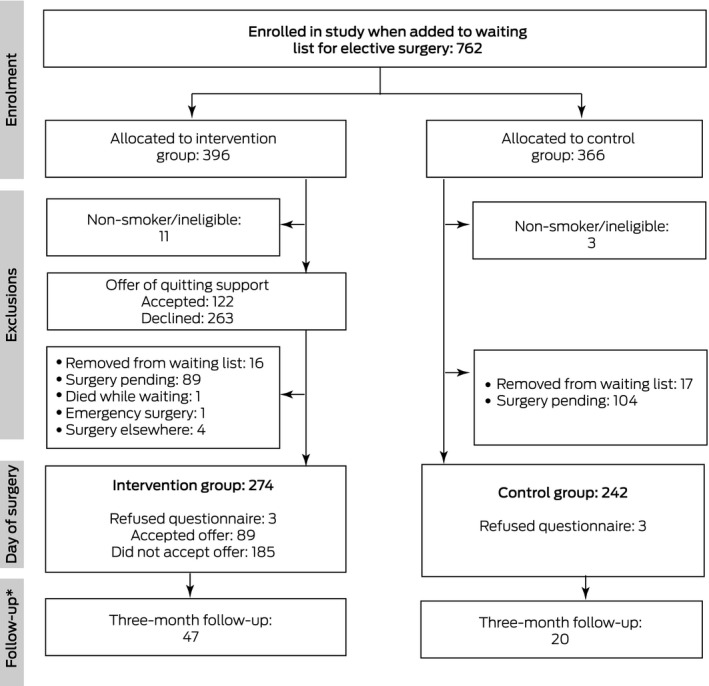

* Participants who had quit smoking at least 24 hours before surgery.

### Intervention group: acceptance and refusal of cessation support

The offer of support was accepted by 122 of 385 people in the intervention group (32%), twelve directly and 110 after we phoned them; 52 of 97 high frequency smokers (54%), 48 of 152 intermediate frequency smokers (32%), and 22 of 105 low frequency smokers (21%) accepted the offer. Of the 263 non‐acceptances, 140 were non‐responses to voicemail messages, and 102 were active refusals (including 23 people with other quitting plans: unassisted quitting, twelve; own NRT, six; varenicline, five) (Box 3).

Box 3 **Contact flowchart and acceptance outcomes for 385 participants in the intervention group**

**Table:** 

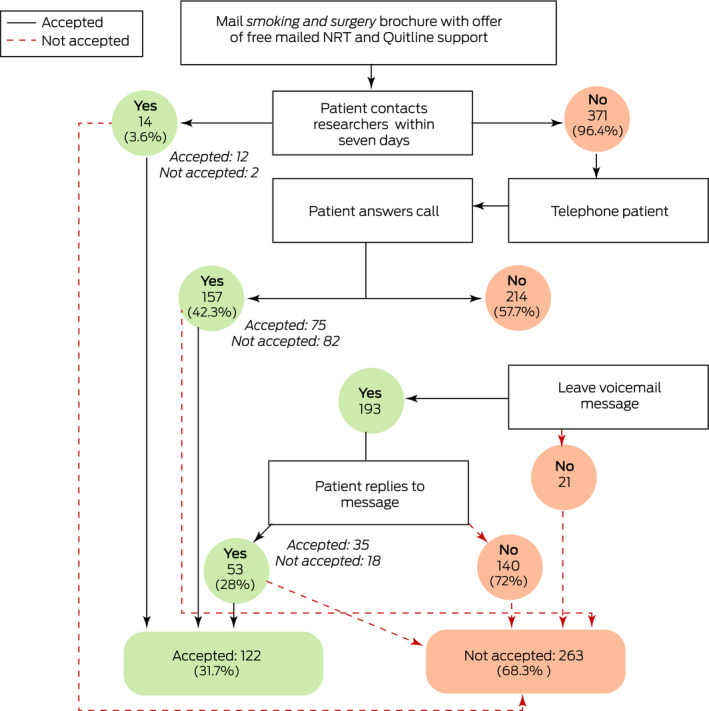

Sex, age, and surgery type distributions were similar for the control and intervention groups. The mean age of participants in the intervention group who accepted help (53 years; standard deviation [SD], 14.6 years) was higher than that of those who did not (49 years; SD, 14.9 years; mean difference, 3.9 years; 95% CI, 0.1–7.6 years). The mean reported number of cigarettes smoked at the time of enrolment, as reported on the day of surgery, was 15.6 per day (SD, 9.3) for the participants who accepted support and 11.4 per day (SD, 7.1) for those who did not (difference, 4.2 per day; 95% CI, 2.0–6.4 per day) (Box 4). Thirteen people who accepted help had not completed the health questionnaire item about the amount smoked. Differences in reported smoking in the health questionnaire and on the day of surgery resulted in six people receiving higher dose NRT packs than anticipated and two receiving lower dose packs.

Box 4 **Characteristics reported by participants on the day of surgery**

**Table:** 

		Intervention group		
	Control group	All	Declined support	Accepted support
Participants*	242	274	185	89
Sex (women)	136 (56%)	157 (57%)	106 (57%)	51 (57%)
Age (years), mean (SD)	50.2 (15.2)	50.4 (14.9)	49.1 (14.9)	53.0 (14.6)
Cigarettes per day at time of enrolment, mean (SD)	11.8 (6.6)	12.8 (8.1)	11.4 (7.1)	15.6 (9.3)
Surgery category				
1 (within 30 days)	30 (12%)	29 (11%)	21 (11%)	8 (9%)
2 (within 60 days)	170 (70%)	194 (71%)	129 (70%)	65 (73%)
3 (within 12 months)	42 (17%)	51 (19%)	35 (19%)	16 (18%)

SD = standard deviation.

* Data provided by 510 participants; three participants in each group declined to be interviewed on the day of surgery.

Reported adverse events were not clinically serious, but all but one led to discontinuing NRT (Box 5).

Box 5 **Adverse events reported at five weeks (telephone follow‐up)**

**Table:** 

	Nicotine patch	Nicotine lozenge
Participants contacted	79	105
Localised skin reactions	5	0
Headache	2	1
Nausea	1	6
Dizziness	1	1
Sleep disturbance	2	0
Chest pain	1	0
Mouth/throat irritation	0	6
Reflux	0	2

Registration for the Quitline call‐back service was declined by 29 of those who accepted NRT help (24%); 45 of the 93 people who accepted referral (48%) had a median of two (IQR, 1–3) sessions before surgery, while 48 could not be contacted by Quitline or refused calls. On the day of surgery, 44 of 274 intervention (16%) and three of 242 control participants reported contact with Quitline (1%).

### Cessation of smoking before the day of surgery

On the day of surgery, six of 516 participants declined to be interviewed and were classified as current smokers. Ninety‐eight patients claimed to have quit, but 15 were classified as current smokers (13 with carbon monoxide values in the range 9–33 ppm, two who refused testing). Quitting at least 24 hours before surgery was more likely for intervention group participants, both before (OR, 2.05; 95% CI, 1.32–3.17) and after excluding twelve participants for whom carbon monoxide testing could not be undertaken (*post hoc* analysis [Supporting Information, part 1]: OR, 1.97; 95% CI, 1.22–3.15). Quitting at least four weeks before surgery was more likely for intervention participants (OR, 2.20; 95% CI, 1.08–4.50), as was quitting activity during the waiting period (OR, 1.56; 95% CI 1.17–2.09) (Box 6). Quitting before surgery was not influenced by sex (data not shown).

Box 6 **Smoking status reported by participants on the day of surgery**

**Table:** 

Outcome	Control group	Intervention group	Odds ratio (95% CI)
Participants	242	274	
Quit smoking at least 24 hours before surgery*	25 (10%)	58 (21%)	2.05 (1.32–3.17)
Verified by carbon monoxide testing	22 (9%)	49 (18%)	1.97 (1.22–3.15)
Quit smoking at least four weeks before surgery*	10 (4%)	25 (9%)	2.21 (1.08–4.50)
Attempted to quit during wait for surgery^†^	53 (22%)	94 (34%)	1.56 (1.17–2.09)
Used medications for quitting	29 (12%)	95 (35%)	2.89 (1.98–4.22)

* Includes twelve people (control, three; intervention, nine) for whom carbon monoxide testing could not be undertaken.

^†^ Attempts sustained for more than 24 hours.

Twenty‐nine people in the control group (12%) and 95 in the intervention group (35%) used cessation medications during the waiting period (Box 6). In the intervention group, 23 people who had accepted support (26%) and 35 who had declined support (19%) had quit smoking at least 24 hours before surgery, as had 25 of 242 people in the control group (10%; intervention/support accepted *v* control: OR, 2.50; 95% CI, 1.49–4.17; intervention/support declined *v* control: OR, 1.83; 95% CI, 1.13–2.94). Quitting was more likely for all participants using cessation medication during the waiting period than for those who did not (29% *v* 12%; OR, 2.5; 95% CI, 1.7–3.6) (Box 7).

Box 7 **Reported smoking outcomes on the day of surgery, by cessation pharmacotherapy use while waiting for surgery**

**Table:** 

		Intervention group	
Outcome	Control group	Declined support	Accepted support
Participants	242	185	89
Cessation pharmacotherapy while waiting	29 (12%)	29 (16%)	66 (74%)
Quit smoking	7 [20%]	11 [38%]	18 [27%]
No cessation pharmacotherapy while waiting	213 (88%)	156 (84%)	23 (26%)
Quit smoking	18 [8%]	24 [15%]	5 [20%]

For the participants who had not quit at least 24 hours before surgery, the number of cigarettes smoked declined during the waiting period from 12.7 (SD, 0.6) to 11.1 (SD, 0.5) per day for the intervention group (mean difference, –1.6 [95% CI, –2.4 to –0.8] cigarettes per day) and from 12.0 (SD, 0.5) to 11.4 (SD, 0.5) per day for the control group (mean difference, –0.6 [95% CI, –0.2 to 1.4] cigarettes per day).

### Follow‐up of people who quit before surgery

Three months after surgery, 39 of the 83 participants who had quit at least 24 hours before surgery (47%) reported they had not smoked in the preceding seven days (intervention, 27 of 58 [47%]; control, 12 of 25 [48%]) (Box 6).

## Discussion

Smokers on elective surgery waiting lists at Frankston Hospital were almost twice as likely to quit before surgery if offered mailed NRT and referral to Quitline. As few potential participants were excluded by our naturalistic study design, people with conditions associated with heavier smoking and lower quit rates, such as mental illness and drug and alcohol misuse,^13^ were included. Study participants were not actively seeking treatment for tobacco use or aware that they were participating in a research trial, increasing the generalisability of our findings. The larger proportion of people who quit after assistance was offered is consistent with findings from studies in non‐surgical settings that the offer was the most important of the three elements of brief interventions (ask, advise, offer help) for triggering unplanned cessation attempts.^14^, ^15^

A Canadian study found that mailed NRT (five weeks, patches only) increased abstinence in adult smokers at six months (7.6% *v* control, 3.0%; OR, 2.65; 95% CI, 1.44–4.89).^16^ In our study, verified quitting before surgery was more frequent among intervention participants (at least 24 hours: 18% *v* 9%; at least four weeks: 9% *v* 4%), differences that were larger than in our pilot study (24 hours: 16% *v* 11%; four weeks: 9% *v* 6%).^12^ Key changes from the pilot study — adding a second information brochure and Quitline referral, providing NRT patches as well as lozenges — probably increased the effectiveness of the intervention. Other studies have found that combination NRT is more effective than monotherapy^17^, ^18^ and that adjunctive counselling enhances NRT.^18^

Peri‐operative cessation trials have typically been undertaken during the one to two weeks preceding surgery.^5^ Interventions during the waiting period provide more time for quitting. The larger proportion of intervention than control participants who had quit four weeks or more before surgery is clinically important, as this duration of abstinence is associated with lower post‐operative wound and respiratory complication rates,^1^, ^2^, ^5^, ^19^, ^20^, ^21^ but not shorter periods.^19^ It has been estimated that five patients need to have quit for four weeks to avert one post‐operative complication.^21^

By doubling both short and medium term abstinence before surgery, the outcomes of our intervention compare favourably with those of other low intensity pre‐operative smoking interventions. A meta‐analysis (seven trials, 1141 participants) found that abstinence was 30% higher for intervention groups at surgery (risk ratio, 1.30; 95% CI, 1.16–1.46), but found no effect on cessation at twelve months.^5^ Higher intensity interventions (face‐to‐face or telephone counselling for more than four weeks, with or without cessation medication) achieved much better results (tenfold higher pre‐surgery cessation, threefold higher abstinence at twelve months^5^), but such programs may not be practicable for many health services. The limited effectiveness of our intervention for heavier smokers suggests that they require more intensive programs, but people who smoke less may be effectively managed with mailed NRT and Quitline support.

While the proportion of participants who quit before surgery was largest for intervention group participants who accepted assistance (26%), the proportion for those who declined help (19%) was also greater than for control participants (10%). All intervention group participants received our brochure on the risks for light smokers, as people are often unaware that the amount smoked has only a limited influence on cardiovascular or cancer risk.^22^ Quit rates among people scheduled for surgery are significantly improved by providing printed materials.^10^

Because of the poor response to our print invitations, hundreds of people who declined help nonetheless discussed smoking with research assistants who followed up by phone. Help offers can elicit unplanned quit attempts,^14^, ^15^ as can unsolicited telephone counselling (cold‐calling) by quitlines.^8^ While some people who declined help may have found the proposed help unsuitable, offering it may have moved them to attempt quitting using other means. Further, the mean smoking level was higher for those who accepted assistance than for those who declined, perhaps partly explaining why the proportion of people who quit after declining help was as high as it was.

Each element of our intervention — the extra brochure, the phone call, mailed NRT, the Quitline referral — could be important for the success of individual smokers. Our intervention could be integrated into routine peri‐operative care in a sustainable, cost‐effective manner.^23^ Partnering with Quitline allowed our health service to receive support from an existing program for cessation counselling, as well as design advice for the printed materials. The total cost for each NRT package, including postage, was about $70. In our study, phone calls and administration were undertaken by a part‐time nurse or pharmacist, but pre‐admission nurses, waiting list staff, or dedicated smoking cessation counsellors could also perform these tasks. Similar strategies have been employed in regional Queensland to increase community Quitline and NRT use,^24^ and mailed NRT could be particularly helpful for overcoming geographic and other barriers.

Surgery has been described as a “teachable moment” for smoking cessation.^25^ Quitting may be motivated as much by desires to reduce surgical risks or to comply with surgical advice as to improve long term health. The large proportion of our control group who quit before surgery, and the even larger proportions in the intervention groups, are consistent with this hypothesis. However, teachability window appeared to be brief; only 47% of those who quit before surgery were still abstinent three months later. Resuming smoking after leaving hospital is not uncommon; in a British study, about 40% of people who had quit had relapsed within six weeks and 60% within twelve months of cardiac bypass surgery or myocardial infarction.^26^ Whether participants who quit before surgery actually relapse, or had intended to resume smoking after surgery, requires further investigation.

### Limitations

Our single centre study was terminated early, and 31% of eligible participants had not had the planned surgery for a variety of reasons, including still waiting for surgery, being removed from the waiting list, or having the operation elsewhere. One problem with waiting list‐based research is that waits for surgery can be long; however, the surgical non‐completion proportion for our pilot study, undertaken before the COVID‐19 pandemic, was just 9.2%.^12^ Non‐completion was unlikely to have biased our results, as it affected both study groups similarly. The fact that a few participants revealed to research assistants, who were supposed to be blinded to allocation, that they were using NRT provided by the hospital, was a minor problem; questions about medication use were asked after information on cessation outcomes had already been recorded. Mailed NRT could be impractical in cases of category 1 (urgent) surgery (often cancer or acute vascular surgery) because of time limits. As pre‐operative quitting is particularly frequent among patients undergoing major surgery,^25^ offering immediate cessation help to people scheduled for urgent surgery could be helpful. We did not have the resources to follow up the smokers who did not quit before surgery, but only those whose abstention before surgery had been verified; this, however, increased the robustness of our finding regarding sustained abstinence by these participants.

### Conclusion

Offering mailed NRT and Quitline support was a practical, safe, and inexpensive strategy for encouraging cessation of smoking before elective surgery. Given the thousands of smoking Australians who have elective surgery each year, the individual and public health benefits could be substantial were this strategy to be adopted by other health services. While not replacing the need for clinicians to advise their smoking patients before surgery, a systematic health service program could improve outcomes and reduce variability in the quality of pre‐surgery smoking cessation support.

## Competing interests

No relevant disclosures.

## Supporting information

Supplementary methods
