# Supplementary material for: Offering mailed nicotine replacement therapy and Quitline support before elective surgery: a randomised controlled trial
Source: Med J Aust. 2022 Mar 10;216(7):357–63. doi: 10.5694/mja2.51453 (PMC9314866; doi:10.5694/mja2.51453)
Supplement: Supplementary file 1 — Supplementary methods [file MJA2-216-357-s001.pdf]

The Medical Journal of Australia

Supporting Information

Supplementary methods

This appendix was part of the submitted manuscript and has been peer reviewed.
It is posted as supplied by the authors.

Appendix to: Webb AR, Coward I, Meanger D, et al. Offering mailed nicotine replacement therapy and Quitline support before elective surgery: a randomised controlled trial. *Med J Aust* 2022; doi: 10.5694/mja2.51453.

1. Protocol variations after trial registration

Randomisation

Participants were enrolled in the trial if they ticked the “current smoker” box on the standard hospital pre-surgery health questionnaire. About 12% of smokers did not complete the subsequent question about the amount smoked (cigarettes per day). The Peninsula Health Human Research Ethics Committee had approved waiver of consent at enrolment and it was not possible to ask participants for this information at the time of listing. The planned randomisation scheme was 1:1 control:intervention, stratified by smoking frequency (low, <10 cigarettes/day; medium, 10–15 cigarettes/day; high, >15 cigarettes/day). Stratification was not undertaken, as this allowed allow inclusion of people who did not respond to this question; this change was made before enrolment commenced.

Expired carbon monoxide assessment

In March 2020, carbon monoxide verification of non-smoker status was halted by Frankston Hospital to mitigate the risk of severe acute respiratory syndrome coronavirus 2 (SARS-CoV-2) transmission, and restrictions were placed on elective surgery volumes; from July 2020, elective surgery ceased completely for several weeks following a hospital SARS-CoV-2 cluster. Several hundred hospital staff were furloughed and research staff redirected to clinical duties. Because of these restrictions, and the fact that it was unlikely that the 30% of randomised participants who had not yet undergone surgery would do so in the foreseeable future, the study was terminated in August 2020. At this point, the numbers of participants who had undergone surgery exceeded the number required according to our power analysis. We therefore added a *post hoc* analysis that included twelve people who had quit smoking but for whom carbon monoxide verification was not possible. As the proportion of people who had misreported their smoking status was 15% (15 of 98 claimed cessations), we deemed it important to include these participants in a separate analysis to avoid underestimating the effectiveness of the intervention.

2. Intervention group: printed invitation card

What can I do to make my **surgery safer?**

Peninsula Health is offering a **free quit smoking** program during 2019. Quitting smoking lowers your risk of serious problems during and after surgery. Staying quit after surgery is one of the best ways to improve your health.

This project has been approved by the Peninsula Health Human Research Ethics Committee.

HREC/4984/PH-2019. Version 1. 7/2/2019

Quitting smoking before surgery: one of the best decisions you could make

- **What is the program?** You contact us and we mail you free medication to help you stop smoking. When you contact us, we will ask you a few questions to decide the right medication for you. This may be nicotine lozenges, patches, or both; enough for about 5 weeks. We suggest you start quitting as soon as you get the package. A Quit Specialist can also call to talk to you about your smoking and offer practical advice and help suited to your needs.
- **What do I do to get the package?** Text YES to our team on **0423 174 473**, or call this number to talk (you can leave a message at any time). If you prefer, email safersurgery@phcn.vic.gov.au to get the package.
- **Who can get the free quit help?** You must be an adult smoker, waiting to have elective surgery at Peninsula Health. Even if you don't yet know the date of your surgery, you're welcome to contact us.

Peninsula Health

Quit Victoria

Heart Foundation

STOP smoking
for safer surgery

Did you know?

- Smoking increases the risk of **complications** with anaesthesia
- Smoking increases your chance of a wound **infection** after surgery
- Smoking **slows healing** after surgery

Peninsula Health

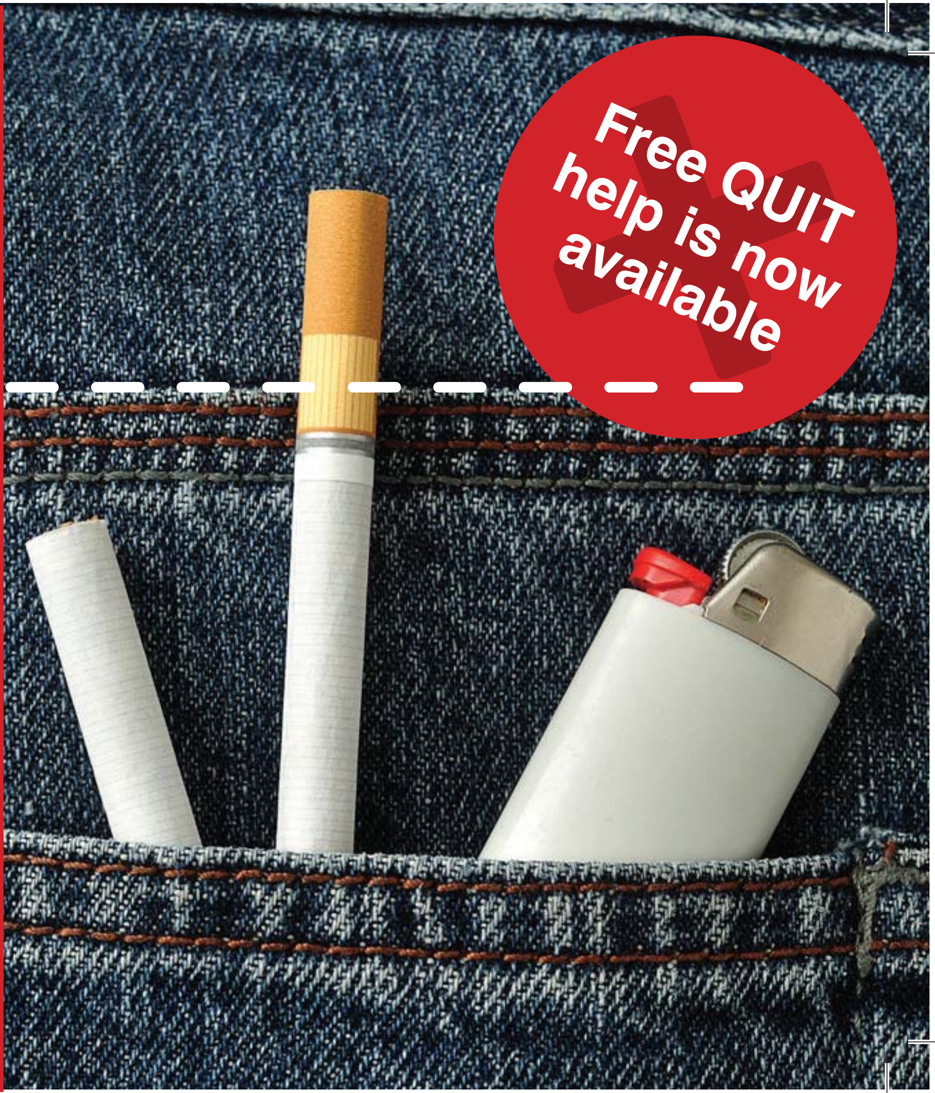

3. Intervention group: additional mailed material for light and intermittent smokers

Every cigarette damages your body.

Cutting down is **not** the solution.

Surgery is a great time to **quit for good**.

To be contacted for quit help before surgery:

text **YES** to **0423 174 473**, or call that number to talk to a team member (you can leave a message at any time).

If you prefer, email
safersurgery@phcn.vic.gov.au

This project has been approved by the Peninsula Health Human Research Ethics Committee.

HREC/4984/PH-2019. Version 1. 7/2/2019

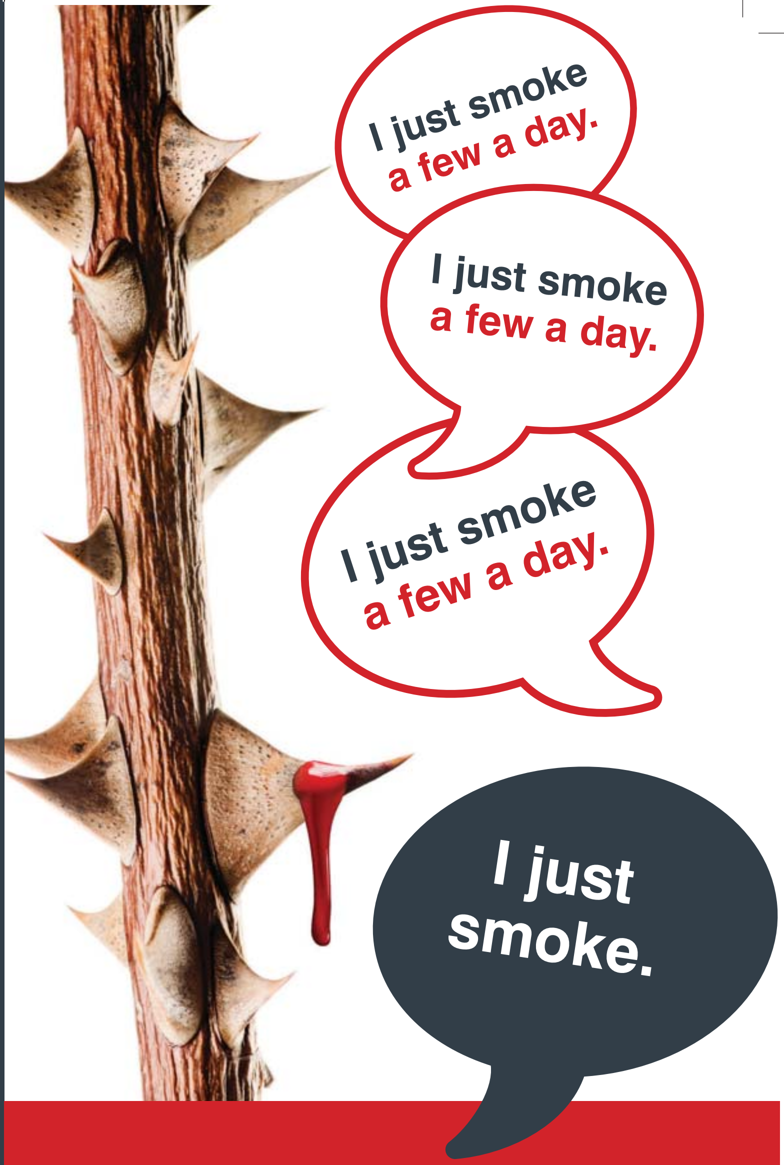

A few a day **hurts you more** than you think.

Peninsula Health

Quit Victoria

Heart Foundation

Stop smoking for safer surgery

Peninsula Health

I just smoke a few a day. That won't really hurt my health…

✕ FALSE

There is **no safe** level of smoking. Every cigarette increases your risk of heart disease, stroke, lung cancer and lung disease.

- If you smoke **just one cigarette** per day, you still have a **much higher risk** of **heart disease** and **stroke** than someone who doesn't smoke (about half the risk of a person who smokes a pack of 20 cigarettes per day). This is because your blood and blood vessels react badly to even small amounts of tobacco smoke. It's why most deaths caused by second-hand smoke are due to heart disease.

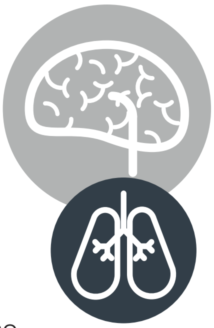

- If you smoke **less than 10** cigarettes per day, you have **16 times** the risk of dying of **lung cancer** than someone who has never smoked.
- You also have **20 times** the risk of developing crippling lung diseases that slowly take your breath (and energy) away, including emphysema.

I'm just a social smoker and I don't smoke every day. That couldn't hurt my recovery from surgery…

✕ FALSE

It is important for everyone to **quit** for as long as they can **before surgery**. Your lungs and blood vessels are delicate and need to be in good shape to help your body cope with the stress of surgery and to heal quickly afterwards.

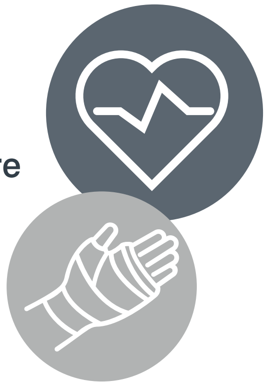

- Smoking reduces the **oxygen supply** to your heart and body.
- Toxins in smoke inflame your lungs and increase your risk of **breathing difficulties** during and after surgery. They also mess with your immune system, increasing your risk of infection.
- Chemicals in smoke attack the lining of your blood vessel walls. Smoking makes your **blood thicker**, stickier and more likely to **clot**.
- Smoking can slow down the healing of skin, bones and other body tissues. It narrows the tiny blood vessels in your skin, slowing down **blood supply** to surgical cuts trying to heal. If parts of your body can't get enough blood, they can **become infected** and even die.

4. Day of surgery data collection

Stop for the Op, Stop for life

Day of surgery data collection tool

Participant number

Date:

Verbal consent for survey ☐ YES ☐ NO

Name ____________________

UR number ____________________

Postcode ☐ ☐ ☐ ☐

To be de-identified at conclusion of study

1. Questions about quitting or attempts to quit

A) Have you quit smoking *since* being told you need surgery?

☐ **YES (abstinent now):** ⟶ How long have you quit for?

_____days______weeks_______months

Have you smoked at all in the last week (even a puff)?

1 – NO 2 - YES

☐ **NO**: ⟶ **B) Have you had *a time since* being told you need surgery, when you quit completely, but relapsed?**

1 – NO

2 – YES. If you *tried*, ***when*** did your last attempt end? (circle answer)

1 - Attempt ended within the last week
2 - Attempt ended 1-3 weeks ago
3 - Attempt ended 1-6 months ago
4 - Attempt ended more than 6 months ago

How long did you quit for on that attempt? (circle answer)

1- 1 day or less
2- 2-6 days
3- 1-3 weeks
4- 1-3 months
5- more than 3 months

⟶ **C) Are you trying to quit now by cutting down?** 1 – NO 2 - YES

(If YES to either A, B,C above, go to question 2, otherwise go to question 3)

2. Questions about methods of quitting (or attempts)

A) Help with Medications

Did you use any stop-smoking medication to help you quit (or when you tried to quit?)

1 – NO

2 - YES, Nicotine replacement (Patch, gum etc.)

2a – Patch
2b – Lozenger
2c – Other NRT
2d – Vaping/e-cigaretted
3 - YES, another stop-smoking medicine. Circle (Champix / Zyban / Other

4 - Are you still using a stop smoking medication?

1 – NO
2 – **YES**

B) Help with Other Methods

Have you spoken with Quitline to help you stop smoking?

1- NOT in the last year
2- YES, in the last year   Approximate number of contacts with Quitline ________

Other methods?

1- Apps/Internet
2- Self-help books
3 – Hypnotherapy
4- Other (write type) ____________________________________

3. How many cigarettes do you smoke per day now?______cigarettes

Age: (circle) Under 25 25-39 40-54 55-64 65-70 Over 70
Sex: (circle) Male Female
Operation name?_________________________________Cat 1 2 3 (circle)
Is this vascular surgery 1 – NO 2 – **YES**
Pension or Health Care Card 1 – NO 2 – **YES**

4. Participants abstinent for > 24 hours (only), written consent, then C.O. level and offer Relapse Prevention Kit (RPK), 3-month follow up call.

A) Written consent obtained 1 – NO 2 – YES Comments______________

B) C.O. level____________p.p.m

C) Accepted RPK 1 – NO 2 – **YES** **Comments__________________________**

D) 3-month call: Preferred number and time (mornings/afternoons)
